# Mesenchymal Stem Cell Administration in Patients with Chronic Obstructive Pulmonary Disease: State of the Science

**DOI:** 10.1155/2017/8916570

**Published:** 2017-02-20

**Authors:** Shih-Lung Cheng, Ching-Hsiung Lin, Chao-Ling Yao

**Affiliations:** ^1^Department of Internal Medicine, Far Eastern Memorial Hospital, Taipei, Taiwan; ^2^Department of Chemical Engineering and Materials Science, Yuan Ze University, Chung-Li District, Taoyuan City, Taiwan; ^3^Division of Chest Medicine, Changhua Christian Hospital, Changhua, Taiwan

## Abstract

Patients with chronic obstructive pulmonary disease (COPD) have chronic, irreversible airway inflammation; currently, there is no effective or curative treatment and the main goals of COPD management are to mitigate symptoms and improve patients' quality of life. Stem cell based therapy offers a promising therapeutic approach that has shown potential in diverse degenerative lung diseases. Preclinical studies have demonstrated encouraging outcomes of mesenchymal stem/stromal cells (MSCs) therapy for lung disorders including emphysema, bronchopulmonary dysplasia, fibrosis, and acute respiratory distress syndrome. This review summarizes available data on 15 studies currently registered by the ClinicalTrials.gov repository, which used different stem cell therapy protocols for COPD; these included bone marrow mononuclear cells (BMMCs), bone marrow-derived MSCs, adipose-derived stem/stromal cells (ADSCs), and adipose-derived MSCs. Published results of three trials indicate that administering BMMCs or MSCs in the setting of degenerative lung disease is safe and may improve patients' condition and quality of life; however, larger-scale studies are needed to evaluate efficacy. Results of another completed trial (NCT01872624) are not yet published, and eleven other studies are ongoing; these include MSCs therapy in emphysema, several studies of ADSCs in COPD, another in idiopathic pulmonary fibrosis, and plerixafor mobilization of CD117 stem cells to peripheral blood.

## 1. Introduction

Chronic obstructive pulmonary disease (COPD) is the fourth leading cause of death in the world and a major cause of chronic morbidity and mortality. Consequently, COPD imposes substantial and increasing economic and social burdens, which are expected to grow in coming decades due to accumulating exposure to COPD risk factors and population aging [1].

Subcategories of COPD include chronic bronchitis (the obstruction of small airways) and pulmonary emphysema. Chronic bronchitis is characterized by hypersecretion of mucus accompanied by a chronic (more than 3 months in 2 consecutive years) productive cough; infectious agents are a major cause. The main feature of pulmonary emphysema is airflow obstruction due to destruction of the alveolar walls distal to the terminal bronchiole, without significant pulmonary fibrosis. Most COPD patients have chronic bronchitis and pulmonary emphysema, together with mucus plugging; however, the severity varies between individuals [2]. Until now, no effective or curative treatment has been devised; thus, the goal of COPD management is to relieve symptoms and to improve quality of life. Experimental models have aimed to investigate the pathophysiology of pulmonary emphysema and to discover new treatment approaches. Stem cell based therapy is a promising approach with great potential in degenerative lung diseases.

Mesenchymal stem cells (MSCs) are known to give rise to several cell types, including osteoblasts, chondrocytes, adipocytes, stroma cells, and skeletal myoblasts; they can differentiate into endothelial cell types. In preclinical studies, MSC treatment has shown promising results in diverse lung disorders, including emphysema, bronchopulmonary dysplasia, fibrosis, and acute respiratory distress syndrome. Intratracheal administration of MSCs has been shown to reduce tissue destruction in elastase-induced emphysema, through secretion of paracrine factors such as epidermal growth factor [3].

MSCs have been proposed to interfere with inflammation responses and exert immunomodulatory effects [4–6]. MSCs have been found to show profound suppressive effects on immune cells and pathways and recent researches have demonstrated that MSCs suppress lung injury and inflammation in several mouse models of inflammatory and immune-mediated lung diseases [7]. Moreover, MSCs have antifibrotic activity and hold great therapeutic potential for treating pulmonary fibrosis. The most widely studied cell types are bone marrow-derived MSCs (BM-MSCs) and adipose-derived MSCs (AD-MSCs).

The first animal model of rodent MSC administration in COPD was published by Shigemura et al. in 2006 [8, 9]. More and more studies on COPD with MSC therapy were published after 2014. MSC sources included humans, rabbits, rats, guinea pigs, or mice [10]. Two principle delivery routes were systemic delivery and local [11]. In regard to cell based therapy in lung diseases, systemic delivery is usually through vascular route, such as intravenous (IV) infusion. Local delivery introduces cells into the lung via intratracheal (IT) or intrabronchial (IB) instillation. The most used route of administration for COPD therapy is IV infusion. No clinical trials of IT delivery of cells for adult COPD patients are listed on ClinicalTrials.gov (https://www.clinicaltrials.gov) until now. IV infusion provides broader biodistribution and is easy to perform; thus, it is the major route of administration in preclinical and clinical studies for the delivery of various cell types (Hicks and Jolkkonen, 2009). Studies on rodent animal models of COPD have proved that IV injection or IT instillation of rodent BM-MSCs or AD-MSCs were safe and effective in attenuating airway injury by ameliorating airway inflammation and apoptosis and IT instillation of BM-MSCs appeared to be superior to IV injection in reducing alveolar hyperinflation and collagen fiber content in the elastase-induced emphysema models (Antunes et al., 2014; Guan et al., 2013; Katsha et al., 2011; Liu et al., 2016). Findings from these animal studies suggested that IT or IB instillation is a preferred and safer way of MSC administration for the treatment of airway diseases. However, the optimal method of delivery will depend on which mechanism of action of the MSC is being utilized.

In 2006, the International Society for Cellular Therapy (ISCT) published a position paper on defining the minimal criteria for multipotent MSC [12]. In addition, ISCT has published a sequence of MSC review papers in* Cytotherapy* focusing on the issue of immunotherapy, controversies, and safety as well [13–18]. Recently, stem cell-mediated therapeutic strategies and lung regeneration have been thoroughly reviewed by Akram et al. [19]. Important issues of MSC therapy in pulmonary disease, including pathogenesis, mechanisms of action, paracrine effects, plasticity and heterogeneity of MSCs, reactive oxygen species in MSC aging, respiratory tissue engineering, and clinical application, have been reviewed [20–29]. A systematic review and a meta-analysis on the published preclinical studies of MSC administration in the treatment of COPD in animal models have been conducted by Liu et al. [10] and suggest a promising role for MSC administration in COPD treatment. Here, we review the findings of published trials to date and the protocol used by the registered trials, including study design, treatment method (cell type, dose, and delivery route), and what kind of outcome measurements are used.

## 2. Review Protocol and Results

### 2.1. Databases Searches

The Cochrane Central Register of Controlled Trials (CENTRAL), PubMed, Medline (Ovid), EMBASE, and ClinicalTrials.gov were searched up until September 2015. Searches were limited to articles in English and used the terms listed in Table 1. Studies were included if (1) they examined the effects of MSCs on COPD or emphysema in human clinical trials and (2) they were available as full text articles. Relevant studies were also identified from among references of included articles.

### 2.2. Search Results

After excluding duplicates and irrelevant studies, only four published studies fit the inclusion criteria. ClinicalTrials.gov currently lists 15 studies of different MSC preparations for COPD treatment; these include three with published results (NCT01110252, NCT00683722, and NCT01306513) (Table 2), which matched the database search results, one completed trial without published results (NCT01872624), and eleven ongoing (NCT02041000, NCT01559051, NCT02161744, NCT02216630, NCT02135380, NCT02645305, NCT02348060, NCT01758055, NCT01849159, NCT02412332, and NCT01916577).

## 3. Discussion

### 3.1. Published Trials of Stem Cell Therapies in COPD (Table 2)

#### 3.1.1. Bone Marrow Mononuclear Cells in Emphysema (NCT01110252)

Bone marrow-derived-mononuclear cells (BMMCs) have displayed beneficial effects for the treatment of various diseases due to their multipotent effects [28] and the easiness of obtaining them for autologous transplantation. BMMCs can be used on the same day of harvesting with low cost and are not at risk of cell rejection (graft-versus-host disease) [30].

Ribeiro-Paes et al. (2011) conducted the first study in COPD to evaluate the safety of the cell therapy with BMMCs in patients with advanced-stage (stage IV dyspnea) pulmonary emphysema [31]. Stem cell stimulation was achieved by subcutaneous injection of 5 *μ*g/kg of granulocyte colony stimulating factor (G-CSF) 3 days before autologous BMMCs were infused into a medial brachial peripheral vein. G-CSF, a hematopoietic growth factor which stimulates the proliferation and mobilization of bone marrow hematopoietic cells to the peripheral blood, [32–36] has been used in cell therapy procedures in both animal and human models [37–42]. The rationale for the use of G-CSF was according to previous results showing that this drug results in an increase in myeloid progenitor cells and CD34+ cells in the bone marrow [32, 43].

Primary outcome measures were evaluated by pulmonary function tests, which included forced vital capacity (FVC), forced expiratory volume in one second (FEV1), and vital capacity (VC). Secondary outcomes included arterial blood gases test of partial pressure of oxygen (PaO_2_) and partial pressure of carbon dioxide (PaCO_2_). Outcomes measures were evaluated at baseline and 30 days after the procedure.

At 1-year follow-up, no adverse effects of BMMC therapy were observed, and the four advanced COPD patients showed significantly improved quality of life (QoL) as well as a more clinical stable condition. Spirometry results of all patients showed a slight improvement in the first 30 days after the cell therapy procedure. However, after 30 days through 90 days, indicators of pulmonary function tended to decrease, although not to preprocedure baseline values, indicating that the BMMC cell therapy may be beneficial for long-term stimulation of pulmonary regeneration.

Besides the lung function results, analysis of cell concentration and markers from mononuclear cells pool before and after infusion showed that CD133^+^ cells were only evident in patients with the highest number of nuclear cells (NC) and best BMMC recovery. These findings suggest that the bone marrow volume to be collected for the BMMC therapy protocol could be increased.

The use of G-CSF is worth further investigation. In the Ribeiro-Paes et al. (2011) study, G-CSF did not show an effect on the proliferation of cell lines CD34^+^ and CD133^+^, which are possibly involved in the regeneration of damaged tissues [44–46]. Therefore, the authors suggest that since there is no consensus on the possible advantage of G-CSF when cells are attained with direct bone marrow puncture [47], G-CSF can be omitted when adopting a direct bone marrow puncture.

Stessuk et al. (2013) included the same participants and adopted the same protocol of Ribeiro-Paes et al. (2011) but with a longer follow-up period of 3 years [48], which corroborated their earlier study. BMMC therapy seems to be safe and no significant adverse effects were reported. Two in four patients showed improved lung function, with predicted FVC increases from 21% to 36.5% and 34% to 58%, respectively. All patients reported significant improvements in their emotional state and physical abilities.

Improved lung function in these two advanced pulmonary emphysema patients could be explained by paracrine effects and diminished plasma levels of inflammation-associated proteins after BMMC infusion. However, the sample size of this study was too small to draw firm conclusions; therefore, no conclusions can be drawn from these studies except no obvious adverse effects during infusion and no obvious safety issues during a 3-year follow-up. However, the safety issue is still questionable as one of four patients died and no information is presented on this. Larger-scale multicenter studies would be needed to evaluate both the safety and efficacy of BMMC therapy in COPD.

#### 3.1.2. Mesenchymal Stem Cells in COPD (NCT00683722)

Weiss et al. (2013) conducted a Phase II, multicenter, randomized, double-blind placebo-controlled study in the United States, to evaluate the safety and efficacy of intravenous allogeneic MSCs (PROCHYMAL®; Osiris Therapeutics Inc.) for treating moderate to severe COPD [49]. The study randomized 62 patients to either MSC (*n* = 30) or placebo (*n* = 32). Patients received four monthly infusions of MSCs (100 × 10^6^ cells/infusion) with a 2-year follow-up. The primary endpoint was safety, assessed by occurrence of adverse events (AEs), electrocardiography (ECG), and COPD exacerbations; efficacy endpoints included pulmonary function, QoL, COPD exacerbation assessments, and markers of systemic inflammation.

No AEs and nor increase in the frequency of exacerbations was observed during the study. Safety analysis indicated that most AEs were mild to moderate in intensity for both MSC and placebo groups [MSC Group, 56.6%; Placebo Group, 65.6%] and a majority were reported as being unlikely related to the MSC treatment [MSC Group, 19 (63.3%); Placebo Group, 22 (68.8%)]. Concerning efficacy, no differences in pulmonary function tests were detected between the groups. The results demonstrate that the administration of MSCs in patients with moderate to severe COPD appears to be safe. However, MSC treatment did not show beneficial effects in terms of either pulmonary function or QoL. A more effective dosage and treatment schedule may be necessary to evaluate efficacy more accurately.

Interestingly, post hoc analyses of systemic inflammation markers showed a decrease in circulating C-reactive protein (CRP) levels in MSC-treated patients at 1 month after the first infusion in patients with elevated CRP levels at baseline (>4.0 mg/L in 29 of 62 patients; 14 in the MSC group, 15 in the placebo group). This trend persisted throughout the study, suggesting that MSCs might inhibit systemic inflammation in COPD.

#### 3.1.3. Bone Marrow-Derived MSC in Emphysema before and after LVRS (NCT01306513)

Currently, the only treatment available for severe emphysema is lung volume reduction surgery (LVRS) to remove the most destroyed parts of the lungs. LVRS is generally performed in two separate sessions, one for each lung, 10–12 weeks apart. LVRS allows improved ventilation in the less affected areas of the lungs that remain, as demonstrated by postsurgical clinical improvement of lung function and increased survival rates. Delayed wound healing after LVRS is an important clinical issue; patients are at high risk for prolonged air leakage, which may be related to the inflammatory sequelae of emphysema.

Stolk et al. (2016) conducted a Phase I, nonrandomized, nonblinded, prospective study in the Netherlands, to test the safety and feasibility of administering bone marrow MSCs (BM-MSCs) before and after LVRS for severe emphysema [50]. The intervention consisted of two BM-MSCs infusions in 10 patients 1 week apart, 4 and 3 weeks prior to the second LVRS, respectively. The primary endpoints were safety and feasibility: safety was assessed by the occurrence of AEs during the first 3 weeks after infusion, according to World Health Organization (WHO) toxicity criteria; feasibility was determined by the number of expanded MSCs in relation to the amount of autologous bone marrow collected, number of passages required, and time to reach study target dose. Secondary endpoints included the difference in days between postsurgical transpleural air leak in each patient after the first and second surgical intervention, and histological responses in resected lung tissue, which were assessed by immunohistochemistry of inflammatory markers, fibrosis, and repair.

All patients showed stable vital functions in the first 48 hours after both BM-MSC infusions, and no toxicity or symptoms related to the infusions were observed in the first 48 hours and at 3 weeks after the second infusion, the day before the second LVRS. Regarding feasibility, seven patients completed the study protocol. Bone marrow could be aspirated from nine, with a mean volume of 158 ml ± 64 ml, and in eight patients the targeted total MSC number was obtained after three expansion cycles. One patient's bone marrow could not be aspirated; one had very poor expansion of MSCs and withdrew from the protocol; one could not be evaluated histologically and the second surgical procedure could not be conducted due to a persistent air leak after the first LVRS.

Clinically, FEV1 had increased by 390 ml ± 240 ml (*P* = 0.03) from baseline at 12-month follow-up. The body weight of all patients increased significantly, by a mean of 4.6 kg (range 1–10 kg; *P* = 0.016). According to immunohistochemistry, alveolar septa showed a threefold increased expression of the endothelial marker CD31 (*P* = 0.016). Besides, significantly increased CD3^+^ and CD4^+^ T cell counts were observed in randomly selected parenchymal tissue sections. Gene expression analysis showed a trend towards higher mRNA expression of interleukin- (IL-) 10 and tumor necrosis factor-*α* (TNF-*α*) stimulated gene/protein 6 (TSG-6) in biopsy tissue after LVRS and MSC treatment.

These results demonstrate that autologous MSC treatment in severe emphysema is feasible and safe. Increased CD31 expression suggests that the BM-MSC treatment might stimulate microvascular endothelial cells in the most severely affected parts of the lung. Therefore, MSC therapy could be a promising treatment approach for emphysema.

### 3.2. Ongoing Trials of Stem Cell Therapies in COPD

#### 3.2.1. Adipose-Derived Stem Cells Therapy (Table 3)

Among the different types of MSCs, adipose-derived MSCs (ADSCs) are a particularly attractive autologous cell source for various therapeutic purposes. First, ADSCs are easy to isolate and relatively abundant. Besides, ADSCs retain a high proliferation capacity in vitro and have the ability to undergo extensive differentiation into multiple cell lineages. Moreover, ADSCs secrete a wide range of growth factors that can stimulate tissue regeneration. Therefore, the clinical use of ASCs is feasible [51].

Current treatment options for COPD are not able to reverse airflow obstruction and accelerated loss of lung function [Celli, 2004]. ADSCs have high immunomodulating capacity and can ameliorate lung injury by secreting several factors with paracrine effects [52]. It has been proven that ADSCs have beneficial effects in animal models of pulmonary diseases [3, 4, 8, 52].

The stromal vascular fraction (SVF) comprises stromal cells isolated from total fat via ex vivo enzymatic digestion of adipose tissue harvested from the patient's abdomen or another applicable area by tumescent syringe liposuction under local anesthesia. SVF cells are not cultured but are isolated from adipose tissue using a sterile process, including a saline rinse to remove red blood cells, draining, and collagenase digestion, which isolates endothelial cells from adipose tissue. Adipose-derived SVF (AD-SVF) harvested from autologous adipose tissue will be delivered via intravenous infusion and inhalation. The number of cells available for infusion varies depending on the amount of tissue processed and the number of cells obtained. SVF contains multiple cellular components, including stem cells, with both regenerative and anti-inflammatory properties. SVF therapy has shown promise for ameliorating the symptoms of COPD. Preclinical studies have found AD-SVF and ADSCs to be safe and effective treatments for COPD. To our knowledge, there has been no published results of ADSC therapy in COPD patients until now.

Though ADSCs hold great potential for use in stem cell therapy, after transplantation, a complex and hostile environment with local hypoxia, oxidative stress, and inflammation may result in a large amount of cell loss or death of ADSCs. In addition, the stemness properties of ADSCs are influenced by the disease state of the donor [53]. The therapeutic effects of ADSCs may massively decrease as a result of insufficient retention and survival of transplanted cells [54]. Thus, tissue engineering approaches need to be dramatically improved by the addition of adjuncts that increase the proliferation and differentiation of ADSCs. Platelet-rich plasma (PRP), which contains high levels of diverse growth factors that can stimulate stem cell proliferation and cell differentiation in the context of tissue regeneration, has recently been identified as a biological material that could be applied to tissue regeneration [53]. The novel approach of adding PRP to ASCs was shown to have promising benefits in regenerative medicine from preclinical and clinical studies.

Ongoing clinical trials evaluating the safety and efficacy of therapy with autologous AD-SVF, ADSCs, cotransplantation of ADSCs, and PRP in COPD registered on ClinicalTrials.gov website include NCT02041000, NCT01559051, NCT02161744, NCT02216630, NCT02135380, NCT02645305, and NCT02348060. Table 3 summarizes the details of ongoing trials with the ADSC therapy.

*(1) NCT02041000 (Recruiting)*. USA investigators are conducting this study to ascertain whether ADSC treatment is safe and effective in improving the disease pathology of COPD. Safety will be assessed as the occurrence and frequency of AEs during the study procedures and at 6-month follow-up. The St. George Respiratory Questionnaire (SGRQ) is an index developed to rate patients QoL by measuring and quantifying health-related variables of patients with airflow obstruction. The Global Initiative for Chronic Obstructive Lung Disease (GOLD) staging system classifies COPD severity based on patients' degree of airflow limitation according to pulmonary function tests. Exercise capacity is measured as the distance a patient can walk in 6 minutes, six-minute walk test (6MWT).

*(2) NCT01559051 (Recruiting)*. NCT01559051 is a Phase I/II, open-label, nonrandomized, multicenter study to evaluate the safety and efficacy of autologous ADSC transplantation in Mexican GOLD III/IV patients. Efficacy is being accessed by whether the therapy improves functional capacity and QoL at 3-month and 6-month follow-up. Safety will be determined by AEs at 6-month follow-up. 

*(3) NCT02161744 (Recruiting)*. Another Phase I, open-label, nonrandomized, multicenter study, NCT02161744, conducted in USA, aims to assess the safety, tolerability, and efficacy of ADSC therapy. Efficacy is to be evaluated by whether ADSC results in less decrease from baseline in lung function parameters (FEV1, FEV1/FVC, diffusing lung capacity for carbon monoxide, and 6MWT). Patients will be followed for 12 months after receiving a single intravenous SVF infusion.

Standard COPD therapy will not be interrupted during the study. In the lipoaspiration procedure, 100–240 ml of lipoaspirate will be extracted, and SVF was isolated with minimal manipulation and reconstituted in saline solution for intravenous administration. 

*(4) NCT02216630 (Recruiting)*. Similar to NCT01559051 and NCT02161744, NCT02216630 is an open-label, nonrandomized, multicenter study of safety and effects of intravenous liposuction-derived autologous ADSCs in COPD; proposed specific aim is to investigate the immunosuppressive potential of nonmanipulated noncultured SVFs obtained via liposuction. Following liposuction extraction of 100 ml of fat, ADSCs will be isolated and injected intravenously. Safety assessment is by number of AEs and efficacy endpoints comprise an FEV1 decline of ≤30 ml at 12-month follow-up (primary endpoint) and decrease in 6MWD of less than 5% over 1 year (secondary endpoint). 

*(5) NCT02135380 (Unknown Status)*. This Phase I/II, open-label, randomized, multicenter study in India is evaluating the safety and efficacy of ADSC for treating idiopathic pulmonary fibrosis (IPF) and COPD. Despite intense research efforts and numerous clinical trials, there is still no effective alternative to lung transplantation to prolong survival of patients with IPF; however, not all IPF patients are eligible for lung transplantation and a large proportion of patients died while awaiting one. Conventional therapies include combinations of corticosteroids, antioxidants, immunosuppressants, and immunomodulatory antifibrotic agents, which will be discontinued for 20 days before screening. Therefore, a safe, effective, and affordable treatment option is needed urgently and the potential application of ADSC as a safe and novel therapeutic agent in lung diseases including COPD and IPF is of great interest. MSCs with antifibrotic actions offer an excellent resource to treat pulmonary fibrosis. Given the limited clinical information regarding the use of SVF and MSC in IPF, this placebo-controlled comparative study will explore the tolerability and effectiveness of SVF for IPF patients in one treatment arm and ADSC in another. Subjects in the SVF arm will receive a single intravenous dose of autologous AD-SVF and those in the MSC arm three intravenous doses of two million ADSCs per kg body weight, given at weekly intervals. Control subjects will receive standard therapy comprising prednisolone ≤10 mg/day or ≤20 mg on alternate days, immunosuppressants such as cyclophosphamide or azathioprine at a dose of 2 mg/kg/day not exceeding 150 mg/day, antioxidants such as N-acetylcysteine at a dose up to 1800 mg/day, and pirfenidone at dose up to 1200 to 1800 mg/day. 

*(6) NCT02645305 (Recruiting)*. Preclinical data show that COPD is closely related to chronic inflammation. In Vietnam, NCT02645305 aims to use ADSC in the form of nonexpanded culture, which is SVF combined with activated platelet-rich plasma (PRP) used to treat COPD. Both SVF and PRP are autologous sources, obtained from adipose tissue and peripheral blood, respectively. This mixture will be intravenously transfused into 20 COPD patients. The primary endpoint includes the blood serum glutamic oxaloacetic transaminase level and glutamic pyruvic transaminase level, which are elevated with liver damage. The secondary endpoints comprise respiration rate, 6MWT, panic attack rates, and the CRP concentration, which will be evaluated before and 6 and 12 months after treatment. 

*(7) NCT02348060 (Recruiting)*. In USA, NCT02348060 will evaluate the effect of SVF treatment on QoL in individuals with COPD for up to 12 months following SVF treatment. The primary outcome is change from baseline over the course of 12 months using participants' assessment of overall QoL assessed by the Chronic Respiratory Disease Questionnaire-Self-Administered Individualized (CRQ-SAI), which is a seven-point Likert response scale. Mean scores are used for baseline and all interviews up to the 12-month follow-up. Secondary outcomes include participants' assessment of breathing comfort, fatigue, emotional function, and mastery (the ability to control feelings of disease-related fear or panic) from baseline to month 12 using the CRQ-SAI dyspnea subscale, CRQ-SAI fatigue subscale, CRQ-SAI emotional function subscale, and CRQ-SAI mastery subscale, respectively.

### 3.3. Bone Marrow-Derived Mesenchymal Stem Cells (Table 4)

#### 3.3.1. NCT01758055 (Unknown Status)

In Iran, NCT01758055 will evaluate the safety of endobronchial transplantation of autologous bone marrow-derived MSC (BM-MSC) in patients with emphysema. The primary endpoint is accessed by pulmonary function, including FVC, FEV1, and FEV1/FVC at baseline and 1-year follow-up. The secondary endpoint is 6MWT. Other outcome measures include oxygen saturation by oximeter, QoL according to the Medical Outcomes Study Questionnaire Short Form 36 Health Survey (SF-36), diffusing lung capacity for carbon monoxide by body-box device, changes of computed tomography scan, dyspnea score with modified Medical Research Council scale, PaO_2_ and PaCO_2_ from the atrial blood gases test, and infection evaluated by a complete blood count. Patients are given spinal anesthesia. About 120 ml of bone marrow is aspirated from each puncture, and after preparation a single dose of ~60 million autologous MSCs are transplanted by bronchoscopy into the endobronchial lumen of these patients.

#### 3.3.2. NCT01872624 (Completed)

This Phase I, nonrandomized, open-label study recruited patients in Brazil with severe heterogeneous emphysema to evaluate the safety of one-way endobronchial valves combined with BM-MSCs and to investigate the potential of MSC administration to decrease local inflammation related to the one-way valve placements. This study determined the effect on QoL and the systemic inflammatory potential of cell therapy, measured by CRP levels, erythrocyte sedimentation rate, and complete peripheral blood count. Moreover, the study investigated whether this treatment modality modulates other markers of inflammatory response and remodeling.

In the treatment group (*n* = 5), BM-MSC was delivered immediately preceding insertion of one-way endobronchial valves by bronchoscopy. In the placebo group (*n* = 5), patients received treatment with one-way endobronchial valves only, with saline injected prior to valve insertion. This study had a 4-month follow-up period to assess safety, QoL, pulmonary function, and inflammatory status (blood samples for CRP and ESR, CBC in peripheral blood).

#### 3.3.3. NCT01849159 (Recruiting)

A problem with MSC transplants in patients with respiratory failure is accelerated apoptosis of transplanted cells under the influence of proinflammatory cytokines and oxidative stress. Since it is known that preconditioning MSCs under hypoxia increases their survival in hypoxic conditions and the expression of growth factors and anti-inflammatory cytokines, this study will investigate whether growing MSCs in hypoxic medium may be beneficial. A Phase I/II, randomized, placebo-controlled study has been designed in Russia to evaluate the safety and efficacy of intravenous infusions of allogeneic BM-MSCs. The BM-MSC treatment group will receive an intravenous MSC suspension of 200 × 10^6^ cells per 400 ml of physiological saline solution, preconditioned under 1% oxygen. Control patients will receive only 400 ml 0.9% physiological saline solution. Infusions will be performed every 2 months for 1 year. The primary endpoint is safety versus placebo, including mortality, AEs and treatment reactions, and vital signs (pulse rate, arterial blood pressure) at baseline and 2-year follow-up. Efficacy endpoints include lung tissue density measured by computed tomography-densitometry, pulmonary function, and diffusion capacity at 6, 12, and 24 months.

### 3.4. BMMC versus ADSC (Table 5)

#### 3.4.1. NCT02412332 (Enrolling Participants by Invitation)

The main goal is to evaluate the safety of infusing BMMC and/or ADSC, separately or concomitantly in COPD patients. The study cohort comprises 20 patients with GOLD grade 3 COPD, divided into four groups: (1) control; (2) BMMC; (3) ADSC; and (4) BMMC + ADSC, with 5 patients per group. Therapeutic stem cells will be obtained from each patient's own bone marrow or adipose tissue, for infusion via a peripheral vein after preparation, separation, expansion, and quality control. Patients will be followed for 12 months. It is expected that this study will extend knowledge about cell therapy in pulmonary diseases and may represent a significant step towards establishing new therapeutic approaches in COPD treatment.

Control subjects will receive no interventions besides conventional (in-course) treatment. BMMC group patients will undergo bone marrow harvesting surgery to obtain approximately 200 ml of bone marrow from the iliac crest under spinal anesthesia. BMMC will be obtained by Ficoll separation and returned by systemic infusion (1 × 10^8^ BMMC in 30 ml saline). In the ADSC group, liposuction will harvest approximately 50 ml of adipose tissue from the abdominal region under spinal anesthesia. Fat tissue will be cultivated for 3 weeks and ADSC returned by systemic infusion (1 × 10^8^ ADSC in 30 ml saline). In the BMMC + ADSC group, BMMC and ADSC will be returned to patients by systemic infusion (5 × 10^7^ ADSC + 5 × 10^7^ BMMC in 30 ml saline). Three patients in each treatment group will have lung perfusion scintigraphy with technetium to evaluate cell engraftment. The primary outcome is total pulmonary capacity, assessed by whole body plethysmography; the secondary outcome is pulmonary morphology, evaluated by the chest X-ray.

Regarding the adipose cultivation, MSCs with preadipocyte characteristics can be isolated from adipose tissue, propagated in vitro, and induced to differentiate in vitro into multiple lineages when treated with established lineage-specific factors. Sharifi et al. (2012) isolated stem cells from human adipose tissue and cultured, expanded, and examined their stemness by determining their surface CD markers and their ability to differentiate into adipocyte lineage. In Sharifi et al.'s study, adhered cells were cultured for 2-3 weeks and the ADSCs were isolated, cultured, and expanded [55]. Thus, the fat being cultivated for 3 weeks seems to be reasonable in this study.

### 3.5. Plerixafor Mobilization of Autologous CD117 Stem Cells (Table 6)

#### 3.5.1. NCT01916577 (Recruiting)

Plerixafor, an CXCR4-SDF1 antagonist, has been found to be a strong inducer of mobilization of hematopoietic stem cells from the bone marrow to the bloodstream as peripheral blood stem cells [56]. Plerixafor in combination with G-CSF is usually being used for mobilization of hematopoietic progenitor cells. Mobilization is the process by which progenitors are made to migrate from the bone marrow into the bloodstream, thus increasing their numbers in the blood. Mobilization is used clinically as a source of hematopoietic stem cells for hematopoietic stem cell transplantation. Combination of G-CSF with plerixafor increases the percentage of persons that respond to the therapy and produce enough stem cells for transplantation but is ineffective in around 15 to 20% of patients.

CD117, a stem cell growth factor receptor, also known as protooncogene c-Kit or tyrosine-protein kinase Kit, is a cytokine receptor expressed on the surface of hematopoietic stem cells as well as other cell types. Altered forms of this receptor may be associated with some types of cancer [57]. CD117 is a receptor tyrosine kinase type III, which binds to stem cell factor (SCF), a substance that causes certain types of cells to grow. Signalling through CD117 plays a role in cell survival, proliferation, and differentiation. Hematopoietic progenitor cells are normally present in the blood at low levels. Signalling through CD117 has been implicated in mobilization. G-CSF indirectly activates CD117. Direct CD117 agonists are currently being developed as mobilization agents.

NCT01916577, a Phase I, open-label, placebo-controlled, randomized study will investigate whether the drug plerixafor (Mozobil®, Sanofi-Aventis U.S. LLC) will lead to significant mobilization of CD117+ stem cells to the peripheral blood. The study cohorts comprise five healthy controls and 15 patients awaiting lung transplantation, five each with COPD, cystic fibrosis, and pulmonary fibrosis. Plerixafor will be given once, at 240 *μ*g/kg subcutaneously, to all the patients, with blood for flow cytometric analysis for CD117+ peripheral blood cells collected just before the dose and 8 hours afterwards. The efficacy endpoint is the number of circulating CD117+ cells per ml of peripheral blood at baseline and change in peripheral blood CD117+ cells per ml following plerixafor treatment. The safety endpoint comprises the number and the incidence of plerixafor-related AEs and severe AEs for 30 minutes after administration, at 1 week and at 1 year posttreatment.

## 4. Conclusion

The first safety study to evaluate cell therapy with BMMCs in COPD was from Ribeiro-Paes et al. (2011) [31]. Their finding that CD34+ and CD133+, which might relate to the regeneration of damaged tissues, did not show proliferation induced by G-CSF suggests that the application of G-CSF can be omitted with the treatment of BMMCs. Weiss et al. (2013) [49] demonstrated that the MSC treatment did not show beneficial effect in either pulmonary function or QoL, indicating that a more effective dosage and treatment schedule may be needed. A decrease in circulating CRP levels in MSC-treated patients 1 month after the first infusion only in patients with elevated CRP levels at baseline suggests that MSCs might inhibit systemic inflammation in COPD. Stolk et al. (2016) [50] demonstrated that autologous administration of BM-MSCs before and after LVRS for severe emphysema patients is feasible and safe; increased CD31 expression indicates that the BM-MSC treatment may stimulate microvascular endothelial cells in the most severely affected parts of the lung and may therefore be a promising treatment for emphysema.

Ongoing clinical trials of stem cell based therapies in COPD include the ASDC, BM-MSC, and plerixafor mobilization of CD117 stem cells to peripheral blood; ASDC transplantation in COPD seems to be a favored treatment modality (7 of 11 ongoing trials) with considerable therapeutic potential. Although no results of ADSC therapy in COPD are yet published, these ongoing trials will expand our limited knowledge of ADSC for COPD patients. In addition to ADSC, BM-MSC, the most studied therapy in preclinical studies, is being examined in two ongoing clinical trials. These efforts will help to elucidate applications of cell based therapies for COPD and degenerative lung diseases.

Taken together, MSCs are currently being used in clinical trials for the treatment of COPD with varying inclusion criteria (different stages of COPD), route of administration (locally or systemically injected), types of cells (autologous or allogeneic), cell dosage, schedule of transplantation, and outcome measurements (pulmonary function, physical capacity, frequency of exacerbations, and mortality). IV delivery is the most common mode of introduction. The current cell dosage has not elicited a long-term therapeutic effect. Further evaluation of efficacy and safety of systemic and local administration of autologous or allogeneic MSCs in the treatment of COPD will be needed to optimize the timing, dosage, and the route of delivery.

## Figures and Tables

**Table 1:** Search strategies used in each database.

Database	Search strategy	Results
CENTRAL	MeSH descriptor: [Lung Diseases, Obstructive] explode all trees AND MeSH descriptor: [Stem Cell Transplantation] explode all trees	4
PubMed	＇＇Pulmonary Disease, Chronic Obstructive＇＇[Mesh] AND ＇＇Stem Cell Transplantation＇＇[Mesh] AND ＇＇English＇＇[Language] AND ＇＇clinical trial＇＇[Publication Type]	4
Medline (Ovid)	exp ^*∗*^Pulmonary Disease, Chronic Obstructive/AND exp ^*∗*^Mesenchymal Stem Cell Transplantation/AND ＇＇Clinical Trial＇＇ [Publication Type]	16
Embase	obstructive airway disease＇/exp/mj AND ＇stem cell transplantation＇/exp/mj AND ([controlled clinical trial]/lim OR [randomized controlled trial]/lim) AND [English]/lim	9
ClinicalTrial.gov	Advanced search with ＇＇Lung Diseases, Obstructive＇＇ for Conditions and ＇＇stem cell＇＇ for Interventions	34
Author search	From references of included articles	1^*∗*^

^*∗*^ Krampera et al., 2006 [6].

**Table 2:** Published trials of stem cell therapies in COPD.

Registry code	NCT01110252	NCT00683722	NCT01306513
Patients (*n*)	Stage III/IV COPD, advanced emphysema (*n* = 4)	Stage II/III COPD (*n* = 62: 30 MSC; 32 placebo)	Stage III COPD, severe emphysema, eligible for LVRS (*n* = 10)

Design	Single center, single arm, open-label, safety study	Multicenter, placebo-controlled, randomized, double-blind, Phase II safety & efficacy study	Single arm, open-label, safety study

Treatment (cells, dose & delivery route)	Autologous BMMC 1 × 10^8^/ml, one IV dose (brachial)	Allogenic MSC 1 × 10^8^/ml, four IV infusions monthly	Autologous BM-MSC 1-2 × 10^6^ cells/kg, two IV infusions 1 week apart, 4 & 3 weeks before 2nd LVRS

Study year (follow-up)	May 2009 (1 and 3 years)	April 2008 (2 years)	October 2010 (1 year)

Primary outcomes	*Lung function*: FVC; FEV1; VC	*AEs* during infusion or by physician/lab assessment *ECGs* during study & follow-up *COPD exacerbations*	*Safety*: AEs ≤ 3 weeks after infusion (WHO criteria) *Feasibility*: quantities of expanded MSCs versus BM collected; passages needed & time to reach target dose

Secondary outcomes	*Arterial blood gases*: PaO_2_; PaCO_2_	*Lung function*: FEV1, FVC, FEV1/FVC, total capacity, DLCO, 6MWT, dyspnea (Borg scale) *QoL*: SGRQ & global assessment *Exacerbations ***:** time to 1st exacerbation; exacerbation rate ratio between study arms *Inflammation markers*: TNF-*α*, IFN-*γ*, IL-2, TGF-*β*, IL-4, IL-5, IL-10, and CRP	Difference (days) between post-LVRS transpleural air leak after 1st versus 2nd LVRS Immunohistochemistry of markers of inflammation, fibrosis, and repair in resected lung tissue

Other markers	*Cell concentration & markers*: NC, BMMC, CD34+, and CD133+	SaO_2_ (before & after 6MWT), CRP, and TGF-*β*	*Clinical*: spirometry, gas transfer, lung volumes, and CT-derived lung densitometry at baseline & 1 year *Immunohistochemistry*: CD3, CD4, CD8, CD31, CD68, Ki-67, or SP-C *Gene expression*: growth factors, immune mediators, proliferation markers, and lung cell markers

Safety results	Safe, no significant AEs	AEs mostly mild to moderate (MSC 56.6%; placebo 65.6%) and unlikely to be procedure-related (MSC 63.3%; placebo 68.8%)	*Safety*: stable vital functions and no change in WHO-toxicity; no infusion-related symptoms *Feasibility*: 7/10 patients completed the study; BM could be aspirated from nine (mean 158 ± 64 ml); target MSC number was obtained with 3 expansion cycles in eight

Efficacy results	Slightly improved lung function ≤30 days after infusion, declined thereafter, but not to baseline Three-year expiratory tests in two patients predicted FVC increase from 21% to 36.5% and 34% to 58%; all patients reported significantly improved emotional and physical status	*COPD exacerbations*: MSC 66.7% versus placebo 46.9% *Median time to 1st exacerbation*: MSC 6.7 months versus placebo not estimated (too few events) *Exacerbation-free at 1 and 2 years*: MSC 46.0% & 31.9% versus placebo 56.3% & 52.7%	*Clinical*: FEV1 rose by 390 ± 240 ml from baseline at 1-year follow-up (*P* = 0.03) Patients' weight significantly increased: mean 4.6 kg (range 1–10 kg; *P* = 0.016) *Immunohistochemistry*: alveolar septa showed tripled expression of CD31 (*P* = 0.016) Significantly higher CD3+ T cell count in alveolar septa after LVRS + BM-MSC versus before (*P* = 0.016); CD4+ T cell count in alveolar septa increased in all but one patient after LVRS + BM-MSC (*P* = 0.30; fold change *P* = 0.047) *Gene expression*: higher mRNA expression of IL10 and TSG6 in biopsy tissue after versus before LVRS + BM-MSC (*P* = 0.06)

Source(s) [reference(s)]	Gupta et al., 2007 [4] Krampera et al., 2006 [6]	Weiss et al., 2008 [7]	Shigemura et al., 2006 [8]

COPD, chronic obstructive pulmonary disease; BMMC, bone marrow mononuclear cell; IV, intravenous; FVC, forced vital capacity; FEV1, forced expiratory volume in 1 second; VC, vital capacity; PaO_2_, partial pressure of oxygen; PaCO_2_, partial pressure of carbon dioxide; NC, nuclear cells; CD, cluster of differentiation; MSC, mesenchymal stem/stromal cell; AE, adverse event; ECG, electrocardiography; DLCO, diffusing lung capacity for carbon monoxide; 6MWT, six-minute walk test; QoL, quality of life; SGRQ, St. George Respiratory Questionnaire; TNF, tumor necrosis factor; IFN-*γ*, interferon *γ*; IL, interleukin; TGF-*β*, transforming growth factor *β*; CRP, C-reactive protein; SaO_2_, peripheral oxygen saturation; LVRS, Lung Volume Reduction Surgery; BM-MSC, bone marrow-derived MSCs; CT, computed tomography; SP-C, surfactant protein-C; WHO, World Health Organization; TSG-6, TNF-*α* stimulated gene/protein 6.

**Table 3:** Ongoing clinical trials with ADSC administration in COPD.

Registry code	NCT02041000	NCT01559051	NCT02161744	NCT02216630	NCT02135380	NCT02645305	NCT02348060
Country	USA	Mexico	USA	USA	India	Vietnam	USA

Start year (follow-up)	January 2014 (6 months)	March 2014 (6 months)	May 2014 (1 year)	August 2014 (1 year)	August 2014 (9 months)	June 2015 (1 year)	November 2015 (1 year)

Status	Recruiting	Recruiting	Recruiting	Recruiting	Unknown	Recruiting	Recruiting

Patients (*n*)	Stage III/IV COPD; age 18–85 years (*n* = 100)	Stage III/IV COPD; age 18–80 years (*n* = 100)	COPD; age ≥ 18 years (*n* = 60)	Stage IIa/III/IV COPD; age 18–85 years (*n* = 200)	IPF, COPD; age 30–70 years (*n* = 60)	Stage IIa/III/IV COPD, age 40–80 years (*n* = 20)	COPD; age ≥ 18 years (*n* = 75)

Design	Multicenter, open-label, nonrandomized safety/efficacy study	Multicenter, open-label, nonrandomized, Phase I/II safety/efficacy study	Multicenter, open-label, nonrandomized, Phase I safety/efficacy study	Open-label, nonrandomized, Phase I/II, safety/efficacy study	Multicenter, open-label, randomized, Phase I/II safety/efficacy study	Open-label, safety/efficacy study	Prospective observational cohort study

Treatment (cells, dose & delivery route)	ADSC	Autologous AD-SVF; IV infusion & inhaled	Autologous AD-SVF; single IV injection	Autologous AD-SVF; IV injection	Autologous AD-SVF/AD-MSC; IV injection	Autologous AD-SVF & PRP; IV injection	Autologous AD-SVF; Single IV injection

Study arm(s) (procedure)	Single arm: ADSC therapy	Single arm: ADSC therapy (lipoaspiration with local anesthesia)	Single arm: ADSC therapy (lipoaspiration & IV SVF saline suspension)	Single arm: ADSC therapy (lipoaspiration)	Randomized: SVF; AD-MSCs; standard therapy (control)	Single arm: ADSC & PRP	Single arm: ADSC therapy (lipoaspiration)

Primary outcomes	*Safety*: AEs occurrence/frequency at follow-up *Efficacy*: SGRQ QoL at follow-up	*Efficacy*: 6MWT at 3 & 6 months *Safety*: number of AEs at 3 & 6 months	*Safety*: frequency of AEs and SAEs at follow-up	*Efficacy*: FEV1 decline of ≤30 mL at follow-up *Safety*: number of AEs at follow-up	*Safety*: treatment emergent AE rates at follow-up	*Safety*: SGOT, SGPT at 1 month	*Efficacy*: CRQ-SAI at 1 year

Secondary outcomes	*Efficacy*: GOLD-classified airflow obstruction & 6MWT at follow-up	*Efficacy*: SGRQ at 3 & 6 months	*Efficacy*: less decrease of FEV1 (ml), FEV1/FVC (%), DLCO (%) & 6MWT (mm) at 6 weeks to 1 year	*Efficacy*: decrease in 6MWD of <5% over 1 year	*Efficacy*: change in predicted FVC (%) & DLCO (%), changes in 6MWT & disease extent and severity (HRCT) at follow-up	*Efficacy*: respiration rate, 6MWT, panic attack rate, and CRP concentration at 1, 6 & 12 months	*Efficacy*: CRQ-SAI dyspnea, fatigue, emotional function, and mastery subscales at 12 months

COPD, chronic obstructive pulmonary disease; ADSC, adipose-derived stem/stromal cell; AE, adverse event; SGRQ, St. George Respiratory Questionnaire; QoL, quality of life; 6MWT, six-minute walk test; IV, intravenous; AD-SVF, adipose-derived stromal vascular fraction; SVF, stromal vascular fraction; SAE, severe adverse event; FEV1, forced expiratory volume in 1 second; DLCO, diffusing lung capacity for carbon monoxide; IPF, idiopathic pulmonary fibrosis; AD-MSC, adipose-derived mesenchymal stem/stromal cell; FVC, forced vital capacity; HRCT, high-resolution computed tomography; PRP, platelet-rich plasma; SGOT, serum glutamic oxaloacetic transaminase; SGPT, serum glutamic pyruvic transaminase; CRP, C-reactive protein; CRQ-SAI, Chronic Respiratory Disease Questionnaire-Self-Administered Individualized.

**Table 4:** Ongoing clinical trials with BM-MSC administration in COPD.

Registry code	NCT01758055	NCT01872624	NCT01849159
Country	Iran	Brazil	Russia

Start year (follow-up)	December 2012 (1 year)	May 2013 (4 months)	March 2014 (2 years)

Status	Unknown	Completed (March 2015)	Recruiting

Patients (*n*)	Moderate to severe emphysema; age 16–70 years (*n* = 12)	Pulmonary emphysema (severe heterogeneous emphysema); age ≥ 18 years (*n* = 10)	Stage III/IV pulmonary emphysema (*n* = 30)

Design	Single arm, open-label, Phase I safety study	Nonrandomized, open-label, Phase I safety study	Randomized, open-label, Phase I/II safety/efficacy study

Treatment	Autologous BM-MSC; single IB injection of 6 × 10^6^ cells by bronchoscopy	Autologous BM-MSC; IB injection by bronchoscopy	Allogeneic BM-MSC; 2 × 10^8^ cells (hypoxic-preconditioned in 1% oxygen); IV infusion every 2 months for 1-year

Study arm(s)	BM-MSC therapy	Valves + BM-MSC treatment (*n* = 5) versus valves + saline controls (*n* = 5)	BM-MSC suspension versus placebo (400 ml 0.9% saline)

Primary outcomes	*Safety*: FVC, FEV1, and FEV1/FVC at 1 year	*Safety*: absence of lung deficits during the procedure and/or follow-up	*Safety*: mortality, AEs & treatment reactions, and vital signs (pulse, arterial blood pressure) at 1 year

Secondary outcomes	*Safety*: 6MWT at 1 year *Others*: oxygen saturation: oximeter test, SF-36 QoL, DLCO, and CT scan; MMRC dyspnea score, blood gases PaO_2_& PaCO_2_, CBC test, at baseline and 1 year	*Safety*: SGRQ *QoL*; *Pulmonary function*: spirometry, flow-volume curve, postbronchodilator test, RV, airway resistance by plethysmography, DLCO & 6MWT *Inflammation*: serum CRP, erythrocyte at 4 months	*Lung tissue density*: CT-densitometry *Pulmonary function*: DLCO, FEV1, TLC, RV, and FEV1/FVC *Physical capacity*: 6MWT *Blood gases*: PaO_2_, PaCO_2_*Serum *IL-6, TNF-*α*, leptin *QoL*: SF-36 *Number and frequency of exacerbationsBody mass index * at follow-up

BM-MSC, bone marrow-derived mesenchymal stem/stromal cell; COPD, chronic obstructive pulmonary disease; IB, intrabronchial; FVC, forced vital capacity; FEV1, forced expiratory volume in 1 second; 6MWT, six-minute walk test; SF-36, Medical Outcomes Study Questionnaire Short Form 36 Health Survey; QoL, quality of life; DLCO, diffusing lung capacity for carbon monoxide; CT, computerized tomography; MMRC, modified Medical Research Council; PaO_2_, partial pressure of oxygen; PaCO_2_, partial pressure of carbon dioxide; CBC, complete blood count; SGRQ, St. George Respiratory Questionnaire; CRP, C-reactive protein; IV, intravenous; AE, adverse event; TLC, total lung capacity; RV, residual volume.

**Table 5:** Ongoing clinical trials comparing BMMC and ADSC therapy in COPD.

Registry code	NCT02412332
Country	Brazil

Start year (follow-up)	April 2015 (1 year)

Status	Enrolling by invitation

Patients (*n*)	Stage II/III COPD; age 40–70 years (*n* = 20)

Design	Randomized, open-label, Phase I/II safety/efficacy study

Treatment	Autologous BMMC/ADSC 1 × 10^8^ in 30 ml saline by IV injection

Study arms & procedures	(1)* Control *(*n* = 5) (2)* BMMC* (*n* = 5): ~200 ml bone marrow will be surgically extracted from the pelvis under spinal anesthesia (3)* ADSC* (*n* = 5): 50 ml of adipose tissue will be extracted by abdominal liposuction under spinal anesthesia (4)* BMMC* + *ADSC* (*n* = 5): 5 × 10^7^ ADSC + 5 × 10^7^ BMMC in 30 ml saline Three patients in each active treatment group will receive lung perfusion scintigraphy with technetium to evaluate cell engraftment

Primary outcomes	*Efficacy: *total pulmonary capacity by whole body plethysmography at 1 year

Secondary outcomes	*Efficacy*: pulmonary morphology (by chest X-ray) and pulmonary function (composite TLC, FVC, FEV1, FEV1/FVC, FEF 25–75, RV, TLC/RV, and airway resistance) at 9 months

BMMC, bone marrow mononuclear cell; ADSC, adipose-derived stem/stromal cell; COPD, chronic obstructive pulmonary disease; IV, intravenous; TLC, total lung capacity; FVC, forced vital capacity; FEV1, forced expiratory volume in 1 second; FEF 25–75, forced expiratory flow at 25–75% of forced vital capacity; RV, residual volume.

**Table 6:** Ongoing clinical trial of plerixafor mobilization of stem cells for COPD therapy.

Registry code	NCT01916577
Country	USA

Start year (follow-up)	August 2013 (1 year)

Status	Recruiting

Patient (*n*)	COPD, cystic fibrosis, pulmonary fibrosis; age 18–70 years; awaiting lung transplant (*n* = 20)

Design	Randomized, open-label, Phase I safety study

Treatment	Autologous CD117+ progenitor cell mobilization; one dose of 240 *μ*g/kg by IV infusion

Study arms & procedures	(1) *Treatment *(*n* = 15): COPD (*n* = 5), cystic fibrosis (*n* = 5), pulmonary fibrosis (*n* = 5) (2) Control (*n* = 5) Blood flow cytometric analysis of CD117+ peripheral blood cells will be collected just before the dose of plerixafor (time zero), and at 8 hours posttreatment

Primary outcomes	*Efficacy*: change from baseline in peripheral blood CD117+ cells per ml at 8 hours posttreatment

Secondary outcomes	*Safety*: number of plerixafor-related AEs/SAEs and number of patients with plerixafor-related AEs/SAEs at 30 minutes, 1 week, and 1 year posttreatment

COPD, chronic obstructive pulmonary disease; IV, intravenous; AE, adverse event; SAE, severe adverse event.

## References

[1] World Health Organization (2007). *Global Surveillance, Prevention and Control of Chronic Respiratory Diseases: A Comprehensive Approach*.

[2] Barnes P. J. (2000). Chronic obstructive pulmonary disease. *New England Journal of Medicine*.

[3] Katsha A. M., Ohkouchi S., Xin H. (2011). Paracrine factors of multipotent stromal cells ameliorate lung injury in an elastase-induced emphysema model. *Molecular Therapy*.

[4] Gupta N., Su X., Popov B., Jae W. L., Serikov V., Matthay M. A. (2007). Intrapulmonary delivery of bone marrow-derived mesenchymal stem cells improves survival and attenuates endotoxin-induced acute lung injury in mice. *Journal of Immunology*.

[5] Iyer S. S., Rojas M. (2008). Anti-inflammatory effects of mesenchymal stem cells: novel concept for future therapies. *Expert Opinion on Biological Therapy*.

[6] Krampera M., Pasini A., Pizzolo G., Cosmi L., Romagnani S., Annunziato F. (2006). Regenerative and immunomodulatory potential of mesenchymal stem cells. *Current Opinion in Pharmacology*.

[7] Weiss D. J., Kolls J. K., Ortiz L. A., Panoskaltsis-Mortari A., Prockop D. J. (2008). Stem cells and cell therapies in lung biology and lung diseases. *Proceedings of the American Thoracic Society*.

[8] Shigemura N., Okumura M., Mizuno S. (2006). Lung tissue engineering technique with adipose stromal cells improves surgical outcome for pulmonary emphysema. *American Journal of Respiratory and Critical Care Medicine*.

[9] Zhen G., Xue Z., Zhao J. (2010). Mesenchymal stem cell transplantation increases expression of vascular endothelial growth factor in papain-induced emphysematous lungs and inhibits apoptosis of lung cells. *Cytotherapy*.

[10] Liu X., Fang Q., Kim H. (2016). Preclinical studies of mesenchymal stem cell (MSC) administration in chronic obstructive pulmonary disease (COPD): a systematic review and meta-analysis. *PLoS ONE*.

[11] Kean T. J., Lin P., Caplan A. I., Dennis J. E. (2013). MSCs: delivery routes and engraftment, cell-targeting strategies, and immune modulation. *Stem Cells International*.

[12] Dominici M., Le Blanc K., Mueller I. (2006). Minimal criteria for defining multipotent mesenchymal stromal cells. The International Society for Cellular Therapy position statement. *Cytotherapy*.

[13] Galipeau J., Krampera M., Barrett J. (2016). International society for cellular therapy perspective on immune functional assays for mesenchymal stromal cells as potency release criterion for advanced phase clinical trials. *Cytotherapy*.

[14] Krampera M., Galipeau J., Shi Y., Tarte K., Sensebe L. (2013). Immunological characterization of multipotent mesenchymal stromal cells—the international society for cellular therapy (ISCT) working proposal. *Cytotherapy*.

[15] Martin I., De Boer J., Sensebe L. (2016). A relativity concept in mesenchymal stromal cell manufacturing. *Cytotherapy*.

[16] Phinney D. G., Sensebé L. (2013). Mesenchymal stromal cells: misconceptions and evolving concepts. *Cytotherapy*.

[17] Phinney D. G., Galipeau J., Krampera M., Martin I., Shi Y., Sensebe L. (2013). MSCs: science and trials. *Nature Medicine*.

[18] Sensebé L., Tarte K., Galipeau J. (2012). Limited acquisition of chromosomal aberrations in human adult mesenchymal stromal cells. *Cell Stem Cell*.

[19] Akram K. M., Patel N., Spiteri M. A., Forsyth N. R. (2016). Lung regeneration: endogenous and exogenous stem cell mediated therapeutic approaches. *International Journal of Molecular Sciences*.

[20] Conese M., Carbone A., Castellani S., Di Gioia S. (2013). Paracrine effects and heterogeneity of marrow-derived stem/progenitor cells: relevance for the treatment of respiratory diseases. *Cells Tissues Organs*.

[21] Conese M., Piro D., Carbone A., Castellani S., Di Gioia S. (2014). Hematopoietic and mesenchymal stem cells for the treatment of chronic respiratory diseases: role of plasticity and heterogeneity. *The Scientific World Journal*.

[22] Griffin M. D., Elliman S. J., Cahill E., English K., Ceredig R., Ritter T. (2013). Concise review: adult mesenchymal stromal cell therapy for inflammatory diseases: how well are we joining the dots?. *Stem Cells*.

[23] Jin Z., Pan X., Zhou K. (2015). Biological effects and mechanisms of action of mesenchymal stem cell therapy in chronic obstructive pulmonary disease. *Journal of International Medical Research*.

[24] Lipsi R., Rogliani P., Calzetta L., Segreti A., Cazzola M. (2014). The clinical use of regenerative therapy in COPD. *International Journal of COPD*.

[25] Mercado N., Ito K., Barnes P. J. (2015). Accelerated ageing of the lung in COPD: new concepts. *Thorax*.

[26] Ojo O., Lagan A. L., Rajendran V. (2014). Pathological changes in the COPD lung mesenchyme—novel lessons learned from in vitro and in vivo studies. *Pulmonary Pharmacology & Therapeutics*.

[27] O'Leary C., Gilbert J. L., O'Dea S., O'Brien F. J., Cryan S.-A. (2015). Respiratory tissue engineering: current status and opportunities for the future. *Tissue Engineering—Part B: Reviews*.

[28] Weiss D. J. (2014). Concise review: current status of stem cells and regenerative medicine in lung biology and diseases. *Stem Cells*.

[29] Yang S.-R., Park J.-R., Kang K.-S. (2015). Reactive oxygen species in mesenchymal stem cell aging: implication to lung diseases. *Oxidative Medicine and Cellular Longevity*.

[30] Silva J. D., Paredes B. D., Araújo I. M. (2014). Effects of bone marrow-derived mononuclear cells from healthy or acute respiratory distress syndrome donors on recipient lung-injured Mice. *Critical Care Medicine*.

[31] Ribeiro-Paes J. T., Bilaqui A., Greco O. T. (2011). Unicentric study of cell therapy in chronic obstructive pulmonary disease/pulmonary emphysema. *International Journal of COPD*.

[32] Slowman S., Danielson C., Graves V., Kotylo P., Broun R., McCarthy L. (1994). Administration of GM-/G-CSF prior to bone marrow harvest increases collection of CD34+ cells. *Progress in Clinical and Biological Research*.

[33] Dicke K. A., Hood D. L., Arneson M. (1997). Effects of short-term in vivo administration of G-CSF on bone marrow prior to harvesting. *Experimental Hematology*.

[34] Isola L. M., Scigliano E., Skerrett D. (1997). A pilot study of allogeneic bone marrow transplantation using related donors stimulated with G-CSF. *Bone Marrow Transplantation*.

[35] Dahl E., Burroughs J., DeFor T., Verfaillie C., Weisdorf D. (2003). Progenitor content of autologous grafts: mobilized bone marrow vs mobilized blood. *Bone Marrow Transplantation*.

[36] Levesque J.-P., Winkler I. G. (2008). Mobilization of hematopoietic stem cells: state of the art. *Current Opinion in Organ Transplantation*.

[37] Frangoul H., Nemecek E. R., Billheimer D. (2007). A prospective study of G-CSF-primed bone marrow as a stem-cell source for allogeneic bone marrow transplantation in children: a Pediatric Blood and Marrow Transplant Consortium (PBMTC) study. *Blood*.

[38] Greinix H. T., Worel N. (2009). New agents for mobilizing peripheral blood stem cells. *Transfusion and Apheresis Science*.

[39] Hokari M., Kuroda S., Chiba Y., Maruichi K., Iwasaki Y. (2009). Synergistic effects of granulocyte-colony stimulating factor on bone marrow stromal cell transplantation for mice cerebral infarct. *Cytokine*.

[40] Pusic I., DiPersio J. F. (2008). The use of growth factors in hematopoietic stem cell transplantation. *Current Pharmaceutical Design*.

[41] Ripa R. S., Jørgensen E., Wang Y. (2006). Stem cell mobilization induced by subcutaneous granulocyte-colony stimulating factor to improve cardiac regeneration after acute ST-elevation myocardial infarction: result of the double-blind, randomized, placebo-controlled stem cells in myocardial infarction (STEMMI) trial. *Circulation*.

[42] Zubair A. C., Malik S., Paulsen A. (2010). Evaluation of mobilized peripheral blood CD34+ cells from patients with severe coronary artery disease as a source of endothelial progenitor cells. *Cytotherapy*.

[43] Johnsen H. E., Jensen L., Gaarsdal E., Hansen P. B., Ersboll J., Hansen N. E. (1994). Priming with recombinant human hematopoietic cytokines before bone marrow harvest expands in vivo and enhances ex vivo recovery of myeloid progenitors in short-term liquid cultures. *Experimental Hematology*.

[44] Jin H., Aiyer A., Su J. (2006). A homing mechanism for bone marrow-derived progenitor cell recruitment to the neovasculature. *Journal of Clinical Investigation*.

[45] Mizrak D., Brittan M., Alison M. R. (2008). CD 133: molecule of the moment. *Journal of Pathology*.

[46] Huertas A., Testa U., Riccioni R. (2010). Bone marrow-derived progenitors are greatly reduced in patients with severe COPD and low-BMI. *Respiratory Physiology and Neurobiology*.

[47] Mehta J. (2005). Cytokines in hematopoietic stem cell transplantation. *Cancer Treatment and Research*.

[48] Stessuk T., Ruiz M. A., Greco O. T., Bilaqui A., Ribeiro-Paes M. J. D. O., Ribeiro-Paes J. T. (2013). Phase I clinical trial of cell therapy in patients with advanced chronic obstructive pulmonary disease: follow-up of up to 3 years. *Revista Brasileira de Hematologia e Hemoterapia*.

[49] Weiss D. J., Casaburi R., Flannery R., LeRoux-Williams M., Tashkin D. P. (2013). A placebo-controlled, randomized trial of mesenchymal stem cells in COPD. *Chest*.

[50] Stolk J., Broekman W., Mauad T. (2016). A phase I study for intravenous autologous mesenchymal stromal cell administration to patients with severe emphysema. *QJM*.

[51] Gentile P., Scioli M. G., Bielli A., Orlandi A., Cervelli V. (2017). Concise review: the use of adipose-derived stromal vascular fraction cells and platelet rich plasma in regenerative plastic surgery. *Stem Cells*.

[52] Schweitzer K. S., Johnstone B. H., Garrison J. (2011). Adipose stem cell treatment in mice attenuates lung and systemic injury induced by cigarette smoking. *American Journal of Respiratory and Critical Care Medicine*.

[53] Tobita M., Tajima S., Mizuno H. (2015). Adipose tissue-derived mesenchymal stem cells and platelet-rich plasma: stem cell transplantation methods that enhance stemness. *Stem Cell Research and Therapy*.

[54] Li Q., Zhang A., Tao C., Li X., Jin P. (2013). The role of SDF-1-CXCR4/CXCR7 axis in biological behaviors of adipose tissue-derived mesenchymal stem cells in vitro. *Biochemical and Biophysical Research Communications*.

[55] Sharifi A. M., Ghazanfari R., Tekiyehmaroof N., Sharifi M. A. (2012). Isolation, cultivation, characterization and expansion of human adipose-derived mesenchymal stem cell for use in regenerative medicine. *International Journal of Hematology-Oncology and Stem Cell Research*.

[56] Cashen A. F., Nervi B., DiPersio J. (2007). AMD3100: CXCR4 antagonist and rapid stem cell-mobilizing agent. *Future Oncology*.

[57] Edling C. E., Hallberg B. (2007). c-Kit—a hematopoietic cell essential receptor tyrosine kinase. *International Journal of Biochemistry and Cell Biology*.

